# Impact of maternal body mass index and gestational weight gain on neonatal outcomes among healthy Middle-Eastern females

**DOI:** 10.1371/journal.pone.0181255

**Published:** 2017-07-17

**Authors:** Tatiana Papazian, Georges Abi Tayeh, Darine Sibai, Hala Hout, Imad Melki, Lydia Rabbaa Khabbaz

**Affiliations:** 1 Laboratoire de Pharmacologie, Pharmacie Clinique et Contrôle de Qualité des Médicaments, Faculty of Pharmacy, Saint-Joseph University of Beirut, Beirut, Lebanon; 2 Department of Nutrition, Faculty of Pharmacy, Saint-Joseph University of Beirut, Beirut, Lebanon; 3 Department of Gynecology and Obstetrics, Hotel Dieu de France Hospital, Ashrafieh, Beirut, Lebanon; 4 Department of Pediatrics, Hotel Dieu de France Hospital, Ashrafieh, Beirut, Lebanon; Universidade de Sao Paulo, BRAZIL

## Abstract

**Background:**

Studies on the relative impact of body mass index in women in childbearing age and gestational weight gain on neonatal outcomes are scarce in the Middle East.

**Objectives:**

The primary objective of this research was to assess the impact of maternal body mass index (BMI) and gestational weight gain (GWG) on neonatal outcomes. The effect of maternal age and folic acid supplementation before and during pregnancy was also examined.

**Subjects and methods:**

This is a retrospective cross sectional observational study of 1000 full term deliveries of women enrolled thru the National Collaborative Perinatal Neonatal Network, in Lebanon. Maternal characteristics such as age, BMI and GWG and neonatal outcomes such as weight, height, head circumference and Apgar score were the primary studied variables in this study. Total maternal weight gain were compared to the guidelines depicted by the Institute of Medicine (IOM).

**Results:**

The negative outcomes of newborns such as lean body weight and macrosomia were significantly present in women who gained respectively below or above the IOM’s cut-off points. Pregestational body mass index influenced significantly the infants’ birth weight, in both the underweight and obese categories. Birth height, head circumference and Apgar score were not influenced by pregestational body mass index or gestational weight gain. No significant associations were found between maternal age and pregestational body mass index and gestational weight gain.

**Conclusion:**

Studies evaluating the impact of weight before and during pregnancy on neonatal outcomes and anthropometrics measurements are lacking in the Middle East. Our results highlight the importance of nutritional counseling in order to shed the extra weights before conceiving and monitor weight gain to avoid the negative impact on feto-maternal health.

## Introduction

Health features have changed across women in child bearing age and are affecting both maternal and neonatal outcomes. Women are entering maternity with a higher body mass index (BMI) and age, hence at a higher initial body weight. On the other hand, obesity, a growing global health problem, is affecting a high percentage of young women, with a negative impact on their own current health and future maternal and neonatal well-being. The 2011 Pregnancy Nutrition Surveillance on maternal health indicators showed respectively a prevalence of 4.5% and 53.7% of women having a prepregnancy BMI in the underweight and overweight category respectively [1]. The Eastern Mediterranean region is not an exception to this epidemic, since statistics highlight an alarming rise of obesity in the Arab world. Data from the Gulf region show gender differences in the prevalence of overweight and obesity, with women having higher rates than men, particularly starting in their mid-20s [2]. Lebanon, a middle income country in the Middle East, is experiencing as well the increased burden of obesity. Two national cross-sectional surveys conducted in Lebanon in 1997 and 2009 depicted an increase of 1.36 kg/m^2^ in the BMI of women aged between 20 and 39 years old [3]. This upward shift of the BMI in this subgroup of Lebanese women in childbearing age surpasses the reported estimate of 0.5 kg/m^2^ in the BMI per decade in woman worldwide [4]. Hence, the repercussion of this rising increase of weight on maternal and neonatal outcomes is essential to be studied.

Weight gain indicators during pregnancy are the result of prepregnancy body mass index (BMI) and gestational weight gain (GWG). During the last decades, researchers studied the influence of those parameters on the development of maternal and neonatal complications, such as gestational diabetes [5], pregnancy induced hypertension [6], macrosomia [7], caesarean [8] and preterm delivery [9]. The main contributors of this excess adiposity are the intake of high energy foods and the sedentary lifestyles. On the other hand, a small percentage of women of child bearing age are underweight and following unbalanced dietary regimes for weight loss, thus predisposing themselves to undernutrition and delivering small-for-gestational age (SGA) infants with an increased risk of mortality and morbidity [10].

In 2009, the Institute of Medicine (IOM) released new guidelines for optimal weight gain during pregnancy, based on the WHO BMI cut-off points, rather than the Metropolitan life insurance tables as used before [11]. This revised version is intended for women of American origin and not applicable to shorter or thinner populations such as Asians; however, it could be applicable to Middle-Eastern women, since they belong to Caucasian origins. This review was necessary since pregestational BMI and GWG had increased worldwide and women’s characteristics had changed regarding their weight and age. Furthermore, those new standards got adopted and used worldwide by obstetricians and midwifes, since they were more specific and based on pregestational BMI, with a narrow weight gain range for obese women.

The principal purpose of this cross-sectional retrospective study was to examine the effect of two anthropometric indicators (pregestational BMI and GWG) on neonatal outcomes in a sample of Lebanese women, since few data on this particular issue are published in the Middle-East and the Arab world. Relationships between maternal age and folic acid supplement intake and neonatal outcomes were also assessed.

## Materials and methods

### Study design

This was an observational retrospective cross-sectional study on data collected by the National Collaborative Perinatal Neonatal Network (NCPNN). This network, established in 1998, is constituted by volunteer professionals, working in different health care institutions in Lebanon. Their major aim is to collect and create a valid database on maternal and neonatal outcomes thru standardized questionnaires, administered in various member hospitals. NCPNN covers around 35% of national births and includes daily perinatal and neonatal data on all deliveries in participating hospitals. This research paper focused on 1000 singleton full-term live births registered to Lebanese mothers aged between 18–40 years, healthy and not suffering from chronic diseases, who delivered after 37^th^ week of gestation in Hôtel-Dieu Hospital (HDF), during the period of 2012–2013. This hospital was selected because it is the university Hospital of Saint-Joseph University and the members of the research team are active working members of this institution. We selected term deliveries to avoid any effect of preterm deliveries on neonatal outcomes. All participants gave their formal written approval before participation. The NCPNN database project was reviewed and approved by the research ethics committee of the American University of Beirut and by the NCPNN representative of HDF.

### Study instrument

A standardized questionnaire designed by NCPNN (S1 and S2 Tables) was administered and completed by research assistants and midwives. It included items that cover parental sociodemographic characteristics, maternal and neonatal outcomes and complications. Face to face interviews were conducted by NCPNN trained research assistants with the participants after delivery and before discharge. All details concerning neonatal outcomes and delivery complications, and maternal anthropometric measurements were recorded directly from the medical records, in order not to end up with irrelevant data. The research team selected from the NCPNN database only the variables of interest with regards to the study purpose. The predictor variables concerning the mothers were age, weight before conception, weight at delivery, height, gestational age at delivery, smoking status, and folic acid supplement intake, weight, height, head circumference, Apgar test at 1 and 5 min, and admission or not to the neonatal intensive care unit were assessed by the medical team upon delivery and recorded by midwives and NCPNN research assistants and constituted the neonatal outcomes variables of this study. Newborns were categorized as low birth weight (LBW) when their weight at birth was less than 2500g, normal when between 2500–4000 g (NBW) and macrosomic when their birth weight was greater or equal to 4000g. Data collection was realized by midwives and NCPNN representatives.

The research team derived two more variables by simple calculations: BMI as defined by weight before conception in kilograms divided by the square of height in meters and GWG as the subtraction between the actual weight at delivery and the initial weight just before becoming pregnant. The BMI classification were categorized according to the WHO cut-off points (underweight<18.5 kg/m^2^, normal weight from 18.5 to 24.9, overweight from 25 to 29.9 and obese > 30 kg/m^2^) [12].

To categorize GWG as below, within or above the recommendations, values were compared to 2009 IOM guidelines, for each prepregnancy BMI category, presented in Table 1 [11]. Women with a GWG within the IOM recommended range were categorized as having a normal GWG, those gaining less or more than the IOM recommendations as having respectively insufficient or excessive GWG.

**Table 1:** Gestational weight gain (GWG) recommendations.

Prepregnancy BMI (kg/m^2^)	Recommended GWG (kg)	Recommended GWG (lb)
Underweight: BMI < 18.5	12.5–18	28–40
Normal weight: 18.5< BMI<25	11.5–16.0	25–35
Overweight: 25<BMI<30	7.0–11.5	15–25
Obese: BMI>30	5.0–9.0	>15

Ref [11]: Institute of Medicine. Weight Gain during pregnancy: reexamining the guidelines, Washington, DC: National Academies Press; 2009

### Statistical analysis

Quantitative variables (age, weight, height, BMI, head circumference and GWG) were evaluated by means and standard deviation analysis. Qualitative variables (smoking status, neonatal outcomes, folic acid supplement use and neonatal complications) were analyzed using a distribution study. The chi-square and the Fisher Exact Tests were used to compare percentages between categorical variables.

Newborns variables that showed associations with p-value <0.20 in univariate analyses were candidates for the multivariate model, according to the Enter method. Collinearity among independent variables was also tested. Independent variables highly correlated were excluded. Multiple logistic regression analyses were conducted and the neonatal outcomes variables included in the model were birth weight, head circumference and birth height. The predictor variables were maternal BMI (reference category was normal BMI), and weight gain (reference category was weight gain within the recommendations of IOM). The confidence interval was adjusted to 95% with a p-value of less than 0.05 as statistically significant. Statistical analyses were performed using the SPSS statistics software version 20.0.

## Results

The general characteristics of the participants are presented in Table 2. One thousand females (mean age of 31.5 years) with a mean BMI of 23 participated in this study. Women were asked by the research team, while filling the NCPNN questionnaire, if they took supplements of folic acid prior to their pregnancy. Surprisingly, only 12% of the participants admitted taking them prior to to getting pregnant. 3% of the participants were active smokers (2.2% and 0.8% cigarette and hookah smokers respectively) and no significant differences were revealed of smoking on neonatal outcomes. However, this result is to be taken with caution, since it was self-reported and pregnant women often hide their true smoking habits.

**Table 2:** General characteristics of the mothers (N = 1000).

**Maternal Age (years) Mean±SD**	31.5±4.4
**Pre-Pregnancy Weight (Kg) Mean±SD**	62.2± 11.0
**Height (cm) Mean±SD**	164.1±5.7
**Pre-Pregnancy BMI (Kg/m**^**2**^**) Mean±SD**	23.0±3.8
**Folic Acid supplementation N(%)**	123(12.3%)
**Smoking N(%)**	30(3%)
**Cigarette Smoking N(%)**	22(2.2%)
**Hookah Smoking N(%)**	8(0.8%)

**Fig 1:**
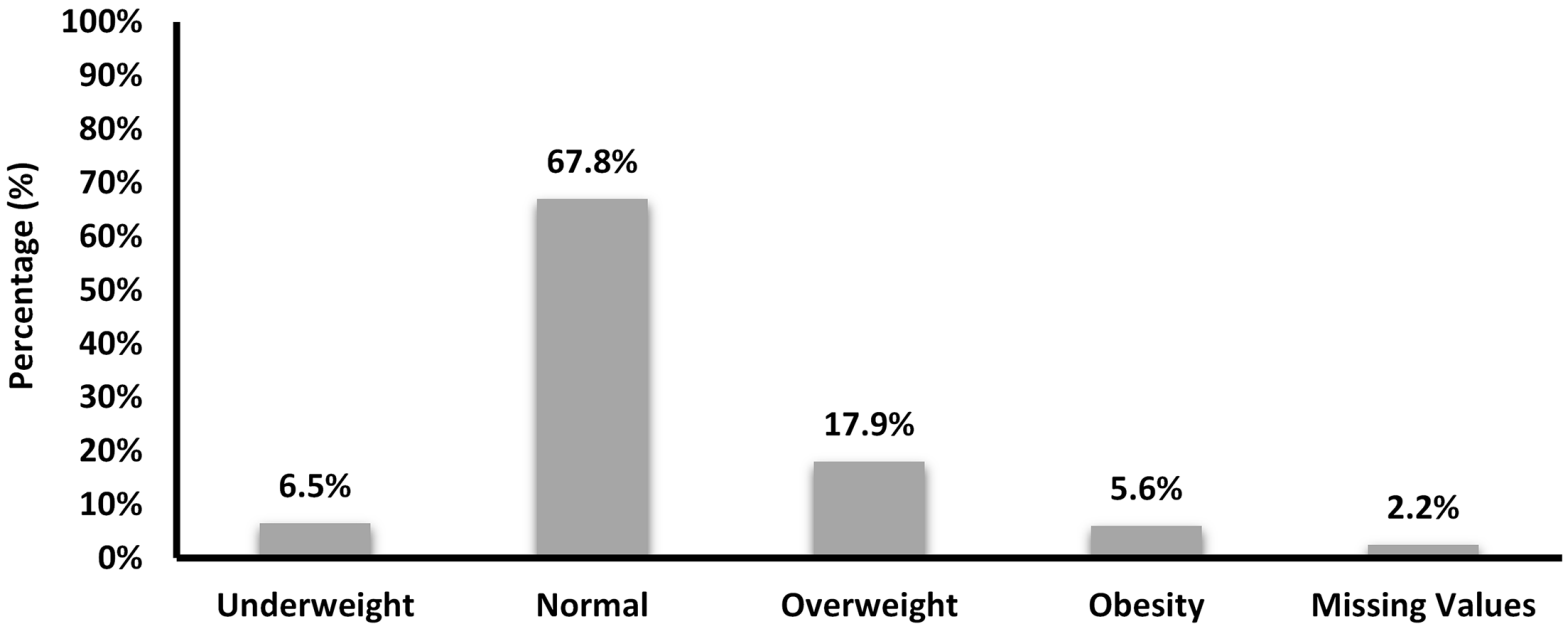
Distribution of mothers according to their pre-pregnancy BMI categories (N = 978).

We stratified women according to their BMI into four groups, as presented in “Fig 1”. Hence, 6.5% of women had a BMI less than 18.5, 67.8% had normal BMI, almost 18% were in the overweight category and 5.6% were obese.

The mean total GWG for all participants was 12.7 kgs +/- 6.0 kgs. Total weight gain was compared to IOM guidelines and presented in Table 3. Overall, 16.1% of women having a normal BMI reached excessive GWG, while 55.4% of overweight and 50.9% of obese women exceeded the IOM cut-off points. It is also worth mentioning that more than one third of women in the underweight BMI category did not reach the recommended margin of GWG.

**Table 3:** Repartition of GWG of mothers according to their BMI (N = 978).

BMI Categories	Mean weight gain ± SD	Adequate weight gain (%)	Insufficient weight gain (%)	Excessive weight gain (%)
**Underweight**	13.30 ± 5.13	33.90%	48.40%	17.70%
**Normal**	13.10 ± 4.34	47.50%	36.40%	16.10%
**Overweight**	12.71 ± 5	37.30%	7.30%	55.40%
**Obese**	10.48 ± 6.31	28.30%	20.80%	50.90%

The average weight, height and head circumference of newborns upon delivery were respectively 3215 grs +/- 389, 49.7 cm +/- 2.1 and 34.5 cm +/- 1.3, indicating that the rate of LBW and macrosomia was respectively 2.55% and 3%. Hence, 94% of newborns fell within the acceptable norms of 2500–4000 grs. In addition, more than 95% of infants had an Apgar score exceeding 7 at 1 and 5 min. All women delivered full term babies, with 2 reported cases of stillborn and 98 admissions to the neonatal care unit because of fetal distress and complications. Detailed results are presented in Table 4.

**Table 4:** General characteristics of infants (N = 1000).

**Weight (g) Mean±SD**	3215±389
**Height (cm) Mean±SD**	49.7±2
**Head circumference (cm) Mean±SD**	34.5±1.3
**Health status of the new born baby**	
**Nursery N(%)**	900(90.0%)
**Intensive Care Unit N(%)**	98(9.8%)
**Stillborn N (%)**	2(0.2%)
**Apgar Score at 1 min <7 N(%)**	45(4.5%)
**Apgar Score at 5 min <7 N(%)**	2(0.2%)

No significant correlations were observed between maternal age and pregestational BMI and GWG with a p value of 0.160 and 0.714 respectively, as presented in Table 5.

**Table 5:** Association between maternal age, BMI & GWG.

	Maternal Age		p-value ^a^
BMI pregestational	< 35 yrs	> = 35 yrs	
**Normal**	523(71.1%)	155(64.0%)	0.160
**Underweight**	49(6.7%)	16(6.6%)	
**Overweight**	124(16.8%)	55(22.7%)	
**Obese**	40(5.4%)	16(6.6%)	
**Total**	736(100.0%)	242(100.0%)	
**Gestational weight gain**			
**Insufficient weight gain**	225(30.9%)	78(32.5%)	0.714
**Normal weight gain**	315(43.2%)	106(44.2%)	
**Excessive weight gain**	189(25.9%)	56(23.3%)	
**Total**	729(100.0%)	240(100.0%)	

BMI, body mass index;

^a^ *p* values were calculated using Chi square test

Table 6 reveals the association between pre-pregnancy BMI and infants’ birth weight; LBW was significantly present (9.0%) in the underweight category of women compared to normal (2.0%), overweight (1.0%) and obese (1.8%), with a p-value less than 0.001. Moreover, macrosomia was more frequently present (16.1%) in the obese category of woman compared to the underweight category of women (0.0%), compared to normal (2.5%) and overweight group (2.0%). However, height, head circumference and Apgar test were not significantly related to pre-pregnancy BMI (p value >0.05).

**Table 6:** Neonatal outcomes by pregestational BMI class.

	Underweight	Normal	Overweight	Obese	p- value^a^^,^^b^
**Birth weight**					< 0.001^a^
**LBW**	6 (9.0%)	14 (2.0%)	2 (1.0%)	1 (1.8%)	
**Normal**	59 (90.1%)	646 (95.4%)	173 (97.0%)	46 (82.1%)	
**Macrosomia**	0 (0.0%)	17 (2.5%)	4 (2.0%)	9 (16.1%)	
**Birth Height**					0.034^b^
**< 48 cms**	15 (23.0%)	74 (11.0%)	19 (11.0%)	7 (12.5%)	
**> 48 cms**	50 (77.0%)	602 (89%0)	160 (89.0%)	49 (87.5%)	
**Head circumference**					0.049^b^
**< 35 cm**	40 (61.5%)	338 (50.0%)	77 (43.0%)	24 (43.0%)	
**≥ 35 cm**	25 (38.5%)	336 (50.0%)	102 (57.0%)	32 (57.0%)	
**Apgar Score**					0.239^a^
**< 7**	5 (11.4%)	30 (68.2%)	5 (11.4%)	4 (9.1%)	
**≥ 7**	60 (6.4%)	647 (69.3%)	174 (18.6%)	52 (5.6%)	

LBW, lean body weight;

^a,b^ *p* values were calculated using the Fisher Exact test(a) and the Chi square test(b)

Results of multivariate logistic regression analysis were conducted are presented in Table 7. A significant positive correlation was found between a pregestational BMI less than 18.5 and low neonatal birth weight and height (p-values of 0.049 and 0.016 respectively). Whereas, in the obese category, macrosomia was significantly correlated to initial high maternal BMI (above 30), with a p-value less than 0.001.

**Table 7:** Multivariate logistic regression analysis of the association between pregestational BMI and neonatal outcomes.

		B	SD	P-value	OR	95% Confidence Interval Limits	
						Lower	Upper
**Underweight**	**Birth weight LBW**	1.093	.555	.049	2.985	1.007	8.850
	**Head circumference<35**	.322	.276	.244	1.380	.803	2.371
	**Small Birth Height**	.800	.331	.016	2.226	1.164	4.255
**Overweight**	**Birth weight**						
	**LBW**	-.545	.781	.485	.580	.125	2.678
	**Macrosomia**	-.251	.567	.658	.778	.256	2.365
	**Head circumference<35**	-.298	.174	.086	.742	.528	1.043
	**Small Birth Height**	.082	.280	.769	1.086	.627	1.879
**Obese**	**Birth weight**						
	**LBW**	-.183	1.085	.866	.833	.099	6.983
	**Macrosomia**	2.007	.462	.000	7.440	3.007	18.409
	**Head circumference<35**	-.335	.289	.248	.716	.406	1.262
	**Small Birth Height**	.280	.436	.520	1.323	.563	3.109
**Reference category: Normal weight**							

LBW, lean body weight; B: unstandardized regression coefficient; SD: standard deviation; OR: odds ratio

Women having a pregestational BMI in the underweight category were respectively 2.985 and 2.226 times more at risk to give birth to an infant of LBW and having a small birth height, compared to women with a normal BMI. In addition, obese women had a 7.44 times more chance to give birth to macrosomic infants, compared to women who had a normal BMI.

The association between GWG and infants’ birth weight was significant (p-value <0.001); LBW was more frequently present among women who gained below IOM recommended GWG, compared to adequate or excessive GWG; whereas macrosomia occurred in 5.7% of women who gained excessive weight during pregnancy. Significant differences were also observed between insufficient GWG and head circumference below 35 cm, among the subgroup of women who gained below IOM recommended GWG. On the other hand, infant’s height was not affected by the GWG of the mothers. Detailed results are presented in Table 8.

**Table 8:** Neonatal outcomes by GWG categories.

	Insufficient GWG	Adequate GWG	Excessive GWG	p- value^a^
**Birth weight**				< 0.001
**LBW**	16 (5.3%)	5(1.2%)	1(0.4%)	
**Normal**	283 (93.3%)	404(96.2%)	230(93.8%)	
**Macrosomia**	4 (1.3%)	11(2.6%)	14(5.7%)	
**Birth Height**				0.001
**< 48 cms**	50(16.6)	50(12.0)	15(6.0)	
**> 48 cms**	251(83.4)	371(88.0)	230(94.0)	
**Head circumference**				< 0.001
**< 35 cm**	171 (57.0%)	208 (49.0%)	97 (40.0%)	
**≥ 35 cm**	130 (43.0%)	212 (51.0%)	147 (60.0%)	

LBW, lean body weight; GWG, gestational weight gain

^a^ *p* values were calculated using the Chi square test

Results of multivariate logistic regression analysis are presented in Table 9. A significant positive correlation was found between insufficient GWG and low neonatal birth weight (p-value of 0.012). Whereas, a negative significant correlation was found between excessive GWG and head circumference and birth height (p-values of 0.050 and 0.032 respectively).

**Table 9:** Multivariate logistic regression analysis of the association between GWG and neonatal outcomes.

		B	SD	P-value	OR	95% Confidence Interval Limits	
						Lower	Upper
**Insufficient GWG**	**Birth weight**						
	***LBW***	1.357	.538	.012	3.884	1.353	11.154
	***Macrosomia***	-.543	.594	.361	.581	.181	1.861
	**Head circumference*<35***	.245	.156	.117	1.277	.941	1.734
	**Small Birth Height**	.309	.222	.163	1.362	.882	2.105
**Excessive GWG**	**Birth weight**						
	***LBW***	-.528	1.116	.636	.590	.066	5.255
	***Macrsomia***	.636	.417	.128	1.888	.833	4.279
	**Head circumference*<35***	-.363	.196	.050	.724	.523	0.999
	**Small Birth Height**	-.686	.319	.032	.503	.269	.941
**Reference category: Normal gain weight**							

LBW, lean body weight; GWG, gestational weight gain;

B: unstandardized regression coefficient; SD: standard deviation; OR: odds ratio

Table 10 presents the effect of folic acid supplementation on neonatal outcomes. Significant associations were observed between the intake of vitamin B9 supplements and infants’ head circumference and Apgar test at 1minute, with p-values of 0.017 and 0.034 respectively. The effect of this supplementation on other neonatal outcomes such as weight and height were not statistically significant.

**Table 10:** Folic acid supplementation and neonatal outcomes.

	Vit B9 supplement intake	No intake of Vit B9 supplement	Total	p-value^a^
**Birth weight**				< 0.268
**LBW**	4 (16.7%)	20 (83.3%)	24 (100%)	
**Normal**	113 (12.3%)	806 (87.7%)	919 (100%)	
**Macrosomia**	6 (20.7%)	23 (79.3%)	29 (100%)	
**Birth Height**				0.263
**< 48 cms**	10 (8.6%)	106 (91.4%)	116 (100%)	
**> 48 cms**	113(13.2%)	742 (86.8%)	855 (100%)	
**Head circumference**				0.017
**< 35 cm**	48 (10.1%)	428 (89.9%)	476 (100%)	
**≥ 35 cm**	**75 (15.2%)**	418 (84.8%)	493 (100%)	
**Apgar Score 1 mn**				0.034
**< 7**	1 (2.3%)	43 (97.7%)	44 (100%)	
**≥ 7**	**122 (13.1%)**	806 (86.9%)	928 (100%)	

LBW, lean body weight; Vit B9, folic acid;

^a^ p values were calculated using the Fisher Exact test

## Discussion

This retrospective cross sectional study examined the association between pregestational BMI, GWG and neonatal outcomes in the Middle East. The study material was derived from NCPNN, whose aim is to build a national database concerning maternal and neonatal outcomes, in order to improve and tackle efficiently problems affecting the health of the mother and the newborn, since it’s the only study dealing with this issue in the Middle East.

GWG is an important predictor of health status on short and long term, in both the mother and the infant. Health consequences vary from low birthweight to macrosomia, gestational diabetes, and obesity in both the mother and the child. Statistics have shown that the majority of pregnant women in US currently exceed the IOM recommendations for GWG [13]. In a large cohort, among 52988 US pregnant women, who gave birth in 2004–2005, 40% of normal weight and 60% of overweight women surpass the IOM range [14]. Similar results were published by Crane et al., in 2009, highlighting the same weight gain phenomena above the IOM recommendations in a sample of Canadian women [15]. Johnson et al studied too this phenomenon in a cohort of 8283 women and recorded that 73% exceed the IOM guidelines [16]. Latest published results in the US, showed that 21% of women gained less than the recommendations, while 47% exceeded the limits set by IOM [17]. In our sample, fortunately only 16% of women in the normal BMI range exceeded the IOM recommendations, however excessive GWG above the norms was alarming in the overweight and obese subgroups, where almost half the participants’ exceeded the limits set by IOM, depending on their initial BMI. This excess GWG implores the necessity of a closer follow-up by the medical team with regular dietetic consultations to prevent the negative outcomes of excessive maternal weight gain on pregnancy and neonatal outcomes.

In our study, GWG below IOM recommendations were associated with higher rates of low birth weight (< 2500g) and infant’s head circumference of less than 35 cm, similar to the results published by Crane et al in 2009 [15]. This issue highlights again the importance of continuous screening and follow-up.

The influence of maternal age on GWG remains controversial. In our sample, women aged above 35 did not have the highest BMI, nor did they achieve excessive GWG, unlike the results published by Chasan-Taber et al., which associated age beyond 30 as being a contributor to weight gain above the IOM recommendations [18]. In another cohort conducted among 1950 women in Australia, a relationship between maternal age and GWG was observed specially in younger women gaining above IOM recommendations, compared to those aged 35 years and above [19]. Nevertheless, Khalil et al. studied the influence of maternal age on adverse pregnancy outcomes and concluded that the age parameter should be combined with other maternal characteristics to achieve a negative impact [20].

In our study, abnormal Apgar scores were not observed in women older than 35 years old or having a high BMI or gaining weight above the recommendations or among pregnant smokers. In addition, smoking status did not influence neonatal outcomes nor the admission to the neonatal intensive care unit, probably because of the small sample of women who confessed being active smokers during their pregnancy. Nevertheless, it is important to pinpoint the low percentage of women (12%) taking folic acid supplements in this large sample, at least one month before getting pregnant. In our study, the impact of the supplementation of this vitamin on neonatal outcomes, showed significant results concerning head circumference and Apgar test. However, those results should be interpreted with caution, since we don’t have any data on their dietary intake, nor the folic acid status of the participants’. In a review published by Uitert E. et al, folic acid supplementation before and during pregnancy was significantly associated with an increase in infants’ birth weight, with no influence on other neonatal parameters [21]. It is worth mentioning that health policies in the Middle-East should implement firm strategies to encourage the intake of vitamin B9 supplements, by women in child-bearing age to avoid neurological deformities and especially that on national level wheat flour is not fortified by this micronutrient, as in the United States.

It is largely proven in the literature that there is a linear correlation between maternal pre-pregnancy BMI and the mean birth weight of the infant [5]. The explanation behind the phenomenon of low birth weight and a BMI less than 18.5 is most likely due to the negative energy balance, the inadequate supply of nutrients from the mother, and the smoking status leading to intrauterine growth retardation. As expected, the mean birth weight of infants of underweight women was significantly lower compared to the other groups, since 2.55% of total deliveries ended up with newborns weighing less than 2500 grs. Even though the result is significant, this percentage is not alarming, probably because all participants delivered after 37^th^ week and mostly (67.8%) had initially a normal BMI. We focused on macrosomia because it has a strong influence on neonatal morbidity and the occurrence of chronic diseases in adulthood [22]. There was a significantly higher rate of macrosomia in the newborns of obese mothers in our sample and this contributes to significant greater challenges at delivery, with increasing risk of shoulder dystocia, C-section and neonatal intensive care unit admissions [23].

Another important finding from our data is the attention to be paid on maternal pre-pregnancy BMI as a major risk factor on neonatal outcomes. According to Krukowski et al., overweight and obese women were respectively 3 and 5 times more likely to achieve excessive gains above the IOM recommendations, with prepregnancy weight status being the strongest predictor [24]. Hence, the importance of starting pregnancy with an acceptable weight, in order to avoid all the metabolic and the delivery complications affecting both the mother and the newborn. However, since half pregnancies are unplanned, overweight and obese women may not have the opportunity to lose weight before conceiving, thus they should be more strictly monitored by their gynecologists’ to minimize the adverse outcomes of gaining excess weight during pregnancy.

Similar to other observational and epidemiological studies, our findings should be considered within the context of the strengths and limitations of our dataset that merit attention. It focused only on deliveries of a single university hospital, HDF, which may not expose a representative picture on national level. Future studies should enroll participants from other hospitals in order to take into consideration regional, educational and social differences. Our results might be influenced by other potential covariates such as parity, education, dietary intake and physical activity, which are major components affecting BMI and GWG, but not taken into account in the NCPNN questionnaires. Hence, the research team is conducting a prospective research among pregnant females to study their nutritional intake and physical activity level and their impact on maternal well-being and neonatal outcomes. The current study evaluated low birth weight (< 2500 g) versus macrosomia (> 4000 g), rather than small and large for gestational age, since the sex of the newborn was not included in the database provided at this stage by the NCPNN. Data on smoking status was collected during the face to face interview with the participants’, who could hide their true smoking habits. In addition, the research team did not have detailed information on the duration and the dosage of folic acid supplementation during the whole pregnancy. Finally, codding errors regarding the use of this standardized database could have occurred.

Nevertheless and despite these limitations, our study has major strength compared to Lahmann’s report of Australia, in which many variables were not taken into account such as maternal height and GWG [25]. Recall biases regarding some variables such as prepregnancy weight and height, maternal and gestational age were minimized since they were taken directly from prenatal and medical records of each patient and then coded in the database.

## Conclusion

The developmental origins of many chronic diseases begin during the embryonic phases due to the interactions between the maternal health status and the genotypic variations, thus the importance of adequate weight of pregnant females to prevent future illnesses of offspring’s. Gaining “too much” or “too little” throughout pregnancy represent another significant danger for both the mother and the baby. In the light of the rising prevalence of overweight and obesity worldwide, it’s not surprising that women of childbearing age are among the first line victims too. Our results revealed significant negative neonatal outcomes when women start their pregnancy with an inadequate BMI; thus, nutritional counseling by health care professionals is crucial to reach an optimal BMI before conception when planning a pregnancy and monitor GWG throughout gestation. Since pregnancy is recognized as an ideal time for education and intervention, pregnant women would be strongly motivated to adopt a healthier lifestyle for the benefit of the fetus.

Future researches will aim at evaluating growth velocity and development of newborns that were included in this study, evaluate maternal postpartum weight retention and childhood obesity and at enrolling a larger sample of women from different Lebanese regions in prospective studies in order as well as studying the impact of new variables such as dietary intake and physical activity, in addition to BMI and GWG on maternal and neonatal outcomes. Collaborations should be planned on national level and with neighboring countries to monitor these aspects related to feto-maternal health, to improve the health outcomes of future generations.

## Supporting information

S1 Table NCPNN questionnaire, English version. (PDF)

S2 Table NCPNN questionnaire, French version. (PDF)
