# Supplementary material for: Impact of maternal body mass index and gestational weight gain on neonatal outcomes among healthy Middle-Eastern females
Source: PLoS One. 2017 Jul 17;12(7):e0181255. doi: 10.1371/journal.pone.0181255 (PMC5513447; doi:10.1371/journal.pone.0181255)
Supplement: S1 Table — (PDF) [file pone.0181255.s001.pdf]

National Collaborative Perinatal Neonatal Network (NCPNN)

Normal Nursery questionnaire (printing version)

Entry Date ____ **Questionnaire Code** ____ / ____

Month of Birth ____ **Center Code** ____

Section I: Socio-Demographics

Mother's admitted class ☐ 1 ☐ 2 ☐ 3

Place of Residence

Area ____ **Province** ____

Area of origin

Mother ____ **Province** ____

Father ____ **Province** ____

Mother's Religion ☐ Moslem ☐ Druze ☐ Christian ☐ Other

Father's religion ☐ Moslem ☐ Druze ☐ Christian ☐ Other

Mother's Age ____ **Father's Age** ____

Mother's Education ☐ Illiterate ☐ Read and Write ☐ Elementary ☐ Intermediate ☐ Secondary ☐ Technical ☐ University (Undergrad.) ☐ University (Grad.)

Mother's Work ◯ No ◯ Yes

Parental Consanguinity ◯ No ◯ Yes **Specify**
☐ 1st cousins (mother & father are cousins)
☐ 2nd cousins (parents are cousins)
☐ More (related, but to a lesser extent)

Relationship ____

Total # of people (excluding current newborn) living in the house? ____

Total # of rooms (excluding kitchen & bathrooms) in the house? ____

Go to Next Tab

Section II: Maternal Obstetric History

Obstetrician ◯ Male ◯ Female

Gravidity ____ **Parity** ____

Abortions, Total ____ **Spontaneous** ____ **Induced** ____ **Living Children** ____

Previous neonatal death (1st 28 days of life) ◯ No ◯ Yes **If yes, number** ☐

Previous c-section ◯ No ◯ Yes **If yes, number** ☐

Previous preterm birth ◯ No ◯ Yes **If yes, number** ☐

Cigarette smoking during pregnancy ◯ No ◯ Yes **If yes, #cig/day** ☐

Arguileh smoking during pregnancy ◯ No ◯ Yes **If yes, #arg/week** ☐

Alcohol drinking during pregnancy ◯ No ◯ Yes **If yes, #glass/week** ☐

Prenatal care ◯ No ◯ Yes **If yes, total # of visits** ☐

Earliest visit ☐ 1st trimester ☐ 2nd trimester ☐ 3rd trimester

Ultrasound Abnormality ☐ No (Normal) ☐ Yes (Abnormal)

Height ☐ **cm** **Weight before pregnancy** ☐ **kg** **Weight at delivery** ☐ **kg**

Section II (continue): Maternal Obstetric History

Maternal chronic conditions

Chronic hypertension ◯ No ◯ Yes **Heart disease** ◯ No ◯ Yes

Asthma ◯ No ◯ Yes **Hypothyroidism** ◯ No ◯ Yes

Epilepsy ◯ No ◯ Yes **Hyperthyroidism** ◯ No ◯ Yes

Anemia ◯ No ◯ Yes **Hemoglobinopathies** ◯ No ◯ Yes

Diabetes Mellitus ◯ No ◯ Yes **If yes, type:** ☐ IDDM ☐ NIDDM

Pregnancy-related complication

Urinary tract infection ◯ No ◯ Yes

Bleeding ◯ No ◯ Yes **If yes** ☐ 1st trimester ☐ 2nd trimester ☐ 3rd trimester
☐ Spotting ☐ Placenta previa ☐ Chronic abruptio

Hypertensive disorders ◯ No ◯ Yes **If yes** ☐ Eclampsia ☐ Preeclampsia

Gestational diabetes mellitus ◯ No ◯ Yes **If yes, did she receive insulin** ◯ No ◯ Yes

Hospitalization during pregnancy ◯ No ◯ Yes **If yes, total number of days** ☐

Medications during pregnancy

Regular folic acid intake at least 1 month prior to pregnancy ◯ No ◯ Yes

Antenatal Steriods ◯ No ◯ Yes **If yes, # of courses** ☐ 1 ☐ 2 ☐ > 2

Anti-coagulants ◯ No ◯ Yes **If yes,** ☐ Heparin ☐ Aspirin ☐ Sintrom

Section III: Delivery Characteristics

Intrapartum fever (>38 C) ◯ No ◯ Yes

Duration of rupture of membranes ☐ **hours**

Intrapartum Group B Streptococcus prophylaxis (Penicillin, Ampicillin, Erythromycin) ◯ No ◯ Yes

Mode of delivery ☐ Normal Vaginal ☐ Operative Vaginal ☐ Cesarean Section

Vaginal birth after C-Section (VBAC) ◯ No ◯ Yes

Indication for c-section

- ☐ Fetal distress/Non reassuring fetal heart/Bradycardia/Deceleration/prolapsed
- ☐ Cephalopelvic Disproportion (CPD) / Dystocia / Failure to progress / unfavorable
- ☐ Presentation (breech, transverse..)
- ☐ Multiple pregnancy
- ☐ Previous c-section / scar in uterus / previous myomectomy
- ☐ Elective c\s (precious infant, IVF, old maternal age, tubal ligation...)
- ☐ Other

Anesthesia ☐ None ☐ 1) Epidural ☐ 2) General ☐ 3) Local ☐ 4) Spinal ☐ Combination of two

Combination 1 ☐ **Combination 2** ☐

Amniotic fluid ☐ Oligohydramnios ☐ Polyhydramnios ☐ Meconium ☐ clear

Section IV: Postpartum Complications

In hospital postpartum complications

Maternal death ◯ No ◯ Yes

Thromboembolic events ◯ No ◯ Yes

Postpartum Hemorrhage (bleeding) ◯ No ◯ Yes If yes, hemorrhage treated with:

Pitocin ◯ No ◯ Yes **Methergine** ◯ No ◯ Yes

Prostaglandins ◯ No ◯ Yes **Hysterectomy** ◯ No ◯ Yes

Go to Next Tab

Section V: ADMISSION & GENERAL CHARACTERISTICS TO THE NORMAL NURSERY

Newborn admission status ☐ NN ☐ Observation

Gestation ☐ Single ☐ Twins ☐ Triplets ☐ Plus

Assisted reproductive technology ◯ No ◯ Yes **If yes, type** ☐ IVF ☐ ET ☐ ICSI ☐ GIFT ☐ ZIFT ☐ IUI

Newborn Sex ◯ Male ◯ Female **If male, circumcision** ◯ No ◯ Yes

Apgars Score 1-min ☐ **/10**

Apgar Score 5-min ☐ **/10**

Gestational Age ☐ **weeks** + ☐ **(0-6) days**

Birthweight ☐ **grams**

Birth FOC ☐ **cm**

Birth Length ☐ **cm**

Admission Temprature ☐ **˚C**

Nutrition

- ☐ Exclusive breastfeeding
- ☐ Exclusive formula feeding

☐ Mixed feeding

Hyperbilirubinemia

Unconjugated hyperbilirubinemia (indirect) ◯ No ◯ Yes

Conjugated hyperbilirubinemia (direct) ◯ No ◯ Yes

If hyperbilirubinemia yes, **Phototherapy** ◯ No ◯ Yes

Exchange transfusion ◯ No ◯ Yes

Go to Next Tab

BIRTH DEFECTS

Birth Defects Diagnosed ◯ No ◯ Yes

Cardiovascular

- ☐ Atrial Septal Defect (ASD)
- ☐ Atrioventricular Canal - AV canal
- ☐ Coarctation of the Aorta
- ☐ Dextroposition of the heart
- ☐ Hypoplastic Left Heart Syndrome (HLHS)
- ☐ Malformations of the mitral/aortic valves
- ☐ Malformations of the tricuspid/pulmonary valves
- ☐ Pulmonary Stenosis (PS)
- ☐ Single Ventricle
- ☐ Tetralogy of Fallot (TOF)
- ☐ Transposition of Great Vessels (TGV)
- ☐ Ventricular Septal Defect (VSD)

Gastrointestinal

- ☐ Atresia of esophagus with or without fistula
- ☐ Absence, atresia & stenosis of anus or rectum /imperforated anus
- ☐ Absence, atresia & stenosis of intestine /intestinal obstruction
- ☐ Congenital fistula of rectum and anus
- ☐ Congenital malformation of intestinal fixation
- ☐ Hirschprung's disease / megacolon
- ☐ Liver malformation
- ☐ Macroglossia
- ☐ Meckel's divertulum
- ☐ Pyloric stenosis

Cleft lip & Palate ☐ Cleft lip ☐ Cleft palate ☐ Cleft lip and palate

Neurological

- ☐ Agenesis of corpus callosum
- ☐ Anencephaly
- ☐ Arnold Chiari
- ☐ Dandy walker syndrome
- ☐ Encephalocele
- ☐ Hydrocephalus
- ☐ Spina bifida / Myelomeningocele / Meningocele

Chromosomal ☐ Down's syndrome(T21) ☐ Edward's syndrome(T18) ☐ Patau's syndrome

Respiratory ☐ Choanal atresia ☐ Hypoplasia and dysplasia of lung

Musculoskeletal

- ☐ Clubfoot
- ☐ Congenital hip dislocation or displacia
- ☐ Diaphragmatic hernia
- ☐ Gastroschesis
- ☐ Omphalocele
- ☐ Prune Belly

Urogenital

- ☐ Absent testis
- ☐ Cystic kidney disease
- ☐ Hypospadias
- ☐ Indeterminate sex / ambiguous genitalia
- ☐ Renal agenesis / Potter's syndrome

Other major, or life threatening, birth defect ◯ No ◯ Yes

If please describe in details [ ]

Go to Next Tab

Discharge Characteristics from Normal Nursery

Mode of payment
- ☐ 1) Self ☐ 2) Insurance company ☐ 3) NSSF ☐ 4) MOH
- ☐ 5) HIP ☐ 6) Army / Forces ☐ 7) COOP ☐ 8) Other private
- ☐ 9) Other public ☐ combination of two

Combination 1 [ ] **Combination 2** [ ]

discharge characteristics ☐ Alive ☐ dead

If alive, ☐ Discharged home ☐ Transferred to another hospital **specify** [ ]

Discharged against medical advice ○ No ○ Yes

Newborn discharge weight [ ] **grams**

If dead, Age of death ☐ < 24 hours ☐ 1-7 days

Go to Next Tab

Preterm Labor

1) Preterm labor: ○ No ○ Yes

At what gestational age: [ ] **Weeks**

Cervical dilation [ ] **cm**

2) Was the patient admitted to hospital for preterm labor? ○ No ○ Yes

How many times? [ ]

At what gestational age? [ ] **weeks** **OR** [ ] **months**

3) Was the patient maintained on tocolytics? ○ No ○ Yes

Period on tocolytics:
- ☐ < 1 week ☐ > 1 week and < 1 month
- ☐ > 1 month and less than 2 months ☐ > 2 months

At what gestational age were tocolytics discontinued? [ ] **weeks**

Version 3.0 Last Modified January 2010
