# Supplementary material for: Impact of maternal body mass index and gestational weight gain on neonatal outcomes among healthy Middle-Eastern females
Source: PLoS One. 2017 Jul 17;12(7):e0181255. doi: 10.1371/journal.pone.0181255 (PMC5513447; doi:10.1371/journal.pone.0181255)
Supplement: S2 Table — (PDF) [file pone.0181255.s002.pdf]

Reseau National pour la Collaboration Perinatal Neonatal

Pouponniere (printing)

Date D'entrée ☐ **Code Du Questionnaire** ☐ / ☐

Mois De Naissance ☐ **Centre De Code** ☐

Section I: SOCIO-DEMOGRAPHIE

Classe d'admission de la mère ☐ 1 ☐ 2 ☐ 3

Lieu de Residence

Région ☐ **Mohafaza** ☐

Region d'origine

Mère ☐ **Mohafaza** ☐

Père ☐ **Mohafaza** ☐

Religion de la mère ☐ Musulmane ☐ Druze ☐ Chrétienne ☐ Autre

Religion du père ☐ Musulman ☐ Druze ☐ Chrétien ☐ Autre

Age maternel ☐ **Age paternel** ☐

Education maternelle ☐ Illétrée ☐ Lit/Ecrit ☐ Elémentaire ☐ Complémentaire

☐ Secondaire ☐ Technique ☐ Universitaire ☐ Universitaire (Etudes supérieures)

Travail de la mère durant la grossesse ○ Non ○ Oui

Consanguinité entre mère et père ○ Non ○ Oui **spécifier** ☐ Cousins 1er degré (mère & père sont

☐ Cousins 2nd degré (leurs parents sont cousins)

☐ Autres (parenté éloignée)

Si Consanguinité, citez le lien ☐

Nombre de personnes (excluant le bébé) vivant dans la même maison ☐

Nombre de chambres (excluant cuisine & salles de bain) de la maison ☐

Section II: ANTECEDENTS OBSTETRICAUX DE LA MERE

Obstétricien ○ Homme ○ Femme

Gravidité ☐ **Parité** ☐

Avortements, Total ☐ **Spontanés** ☐ **Induits** ☐ **Enfants vivants** ☐

Morts Néonatales ○ Non ○ Oui **Si Oui, Nombre** ☐

Cesariennes antérieures ◯ Non ◯ Oui **Si Oui, Nombre** ☐

Naissance prématurée précédente ◯ Non ◯ Oui **Si Oui, Nombre** ☐

Cigarettes durant la grossesse ◯ Non ◯ Oui **Si Oui, # cig/ jour** ☐

Arguileh durant la grossesse ◯ Non ◯ Oui **Si Oui, # arg/ Semaine** ☐

Alcool durant la grossesse ◯ Non ◯ Oui **Si Oui, # verre/Semaine** ☐

Soins prénatals ◯ Non ◯ Oui **Si Oui, # total de visites:** ☐

première visite ☐ 1er Trimestre ☐ 2 ème Trimestre ☐ 3 ème Trimestre

Echographie Anormale ☐ Non ☐ Oui

Taille ☐ **cm** **Poids avant la grossesse** ☐ **kg** **Poids à l'accouchement** ☐ **kg**

Section II (continue): Section II: ANTECEDENTS OBSTETRICAUX DE LA MERE

Pathologies chroniques maternelles

Hypertension chronique ◯ Non ◯ Oui **Maladie du cœur** ◯ Non ◯ Oui

Asthme ◯ Non ◯ Oui **Hypothyroïdisme** ◯ Non ◯ Oui

Epilepsie ◯ Non ◯ Oui **Hyperthyroïdisme** ◯ Non ◯ Oui

Anémie ◯ Non ◯ Oui **Hemoglobinopathies** ◯ Non ◯ Oui

Diabète mellitus ◯ Non ◯ Oui **Si Oui, type** ☐ IDDM ☐ NIDDM

Complications obstétricaux

Infection Urinaire ◯ Non ◯ Oui

Saignement ◯ Non ◯ Oui **Si Oui** ☐ 1st trimester ☐ 2nd trimester ☐ 3rd trimester

☐ Traces ☐ Placenta previa ☐ Abruptio chronique

Hypertension ◯ Non ◯ Oui **Si Oui** ☐ Eclampsie ☐ Pre-eclampsie

Diabète Gestationnel ◯ Non ◯ Oui **Si Oui, A-t-elle reçu de l'insuline ?** ◯ Non ◯ Oui

Hospitalization durant La grossesse ◯ Non ◯ Oui **Nbre de jours total** ☐

Medicaments reçus durant la grossesse

Consommation régulière d'acide folique au moins 1 mois avant la grossesse ◯ Non ◯ Oui

Stéroïdes Anténataux ◯ Non ◯ Oui **Si Oui, # de cures** ☐ 1 ☐ 2 ☐ > 2

Anti-coagulants ◯ Non ◯ Oui **Si Oui,** ☐ Héparine ☐ Aspirine ☐ Sintrome

Section III: CARACTERISTIQUES D'ACCOUCHEMENT

fièvre durant l'accouchement (> 38c) ◯ Non ◯ Oui

Durée de la rupture de la poche des eaux ☐ **heures**

Antibiotiques préscrits pour la prévention du streptocoque de groupe B durant l'accouchement ◯ Non ◯ Oui

Naissance par voie basse après césarienne (VBAC) ◯ Non ◯ Oui

Mode d'accouchement ☐ Vaginal (par voie basse) ☐ vaginal assisté ☐ Césarienne

Indications de la césarienne
- ☐ Souffrance fœtale / cœur fœtal allertant/ Bradycardie/ Décélération/ Prolapsus du
- ☐ Disproportion Céphalo-Pelvienne (CPD) / Dystocie / échec de progression / Col de l'utérus
- ☐ Présentation (Siège,transverse,...)
- ☐ Grossesse Multiple
- ☐ Césarienne itérative / Cicatrice au niveau de l'utérus/ Antécédent de
- ☐ Césarienne élective (bébé précieux, FIV, âge maternel avancé, ligature des
- ☐ Autres

Anesthésie ☐ 0) Aucune ☐ 1)Péridurale ☐ 2)Générale ☐ 3)Locale ☐ 4)Rachidienne ☐ 5)Combinaison de 2

Combinaison 1 ☐ **Combinaison 2** ☐

Liquide Amniotique ☐ Oligohydramnios ☐ Polyhydramnios ☐ Méconium ☐ clair

Section IV: COMPLICATIONS POST PARTUM

COMPLICATIONS POST PARTUM

Mort maternelle ○ Non ○ Oui

Evènements thromboemboliques ○ Non ○ Oui

Hémorragie Postpartum (saignement) ○ Non ○ Oui Si oui, hémorragie traitée avec

Pitocine ○ Non ○ Oui **Methergine** ○ Non ○ Oui

Prostaglandines ○ Non ○ Oui **Hystérectomie** ○ Non ○ Oui

Section V: NAISSANCE ET CHARACTERISTIQUES GENERAL- POUPONNIERE

Statut d'admission du nouveau né ☐ Pouponnière ☐ Observation

Grossesse ☐ Unique ☐ Gémellaire ☐ Triplés ☐ Plus

Technologie de reproduction assistée ○ Non ○ Oui **Si oui, type** ☐ FIV ☐ ET ☐ ICSI ☐ GIFT ☐ ZIFT ☐ IUI

Sexe du nouveau né ○ Garçon ○ Fille **Si garçon, Circoncision** ○ Non ○ Oui

Score Apgar 1-minute ☐ **/10**

Score Apgar 5-minute ☐ **/10**

Age gestationnel ☐ **S.A** + ☐ **(0-6) jours**

Poids de naissance ☐ **grams**

Périmètre crânien de naissance ☐ **cm**

Taille de naissance ☐ **cm**

Temp. à l'admission ☐ **˚C**

Alimentation
- ☐ Allaitement exclusif
- ☐ Lait en poudre exclusif
- ☐ Allaitement mixte

Hyperbilirubinémie

Hyperbilirubinémie libre (indirecte): ○ Non ○ Oui

Hyperbilirubinémie conjuguée (directe): ○ Non ○ Oui

Si hyperbilirubinémie "oui", **Photothérapie** ○ Non ○ Oui

Exsanguino-Transfusion: ○ Non ○ Oui

MALFORMATIONS CONGENITALES

Malformations congénitales présentes ○ Non ○ Oui

Cardiovasculaires

- ☐ Communication inter-auriculaire (CIA)
- ☐ Canal Atrioventriculaire (AV canal)
- ☐ Coarctation de l'Aorte
- ☐ Dextrocardie du coeur
- ☐ Hypoplasie ventriculaire gauche
- ☐ Malformations de la valve mitrale ou aortique
- ☐ Malformations de la valve tricuspide ou pulmonaire
- ☐ Sténose Pulmonaire (SP)
- ☐ Ventricule Unique
- ☐ Tétralogie de Fallot (TF)
- ☐ Transposition des Gros Vaisseaux (TGV)
- ☐ Communication inter-ventriculaire (CIV)

Gastrointestinales

- ☐ Absence, atrésie et sténose de l'intestin / obstruction
- ☐ Absence, atrésie et sténose de l'intestin / obstruction
- ☐ Absence, atrésie et sténose de l'intestin / obstruction
- ☐ Fistule congénitale du rectum et de l'anus
- ☐ Malrotation intestinale
- ☐ Maladie de Hirschprung / mégacolon
- ☐ Malformation Hépatique
- ☐ Macroglossie
- ☐ Diverticule de Meckel
- ☐ Sténose du Pylore

Fente labiale et/ou palatine

- ☐ Fente labiale (Bec de lièvre)
- ☐ Fente palatine
- ☐ Fente labio-palatine

Neurologiques

- ☐ Agenèse du corpus callosum
- ☐ Anencéphalie
- ☐ Syndrome de Arnold Chiari
- ☐ Syndrome de Dandy Walker
- ☐ Encéphalocèle
- ☐ Hydrocéphalie
- ☐ Spina bifida / Myelomeningocèle/ Meningocèle

Chromosomique

- ☐ Syndrome de Down (T21)
- ☐ Syndrome d'Edward (T18)
- ☐ Syndrome de Patau (T13)

Respiratoires

- ☐ Atrésie des Choanes
- ☐ Hypoplasie et dysplasie du poumon

Musculo-squelettiques

- ☐ Pieds Bots
- ☐ Luxation congénitale de la hanche
- ☐ Hernie Diaphragmatique
- ☐ Gastroschisis
- ☐ Omphalocèle
- ☐ Syndrome de Prune Belly

Urogénitales

- ☐ Absence testiculaire
- ☐ Reins kystiques
- ☐ Hypospadias
- ☐ Sexe indéterminé
- ☐ Agenèse des reins / Syndrome de Potter

Autres malformations majeurs, ou critique ○ Non ○ Oui

Si oui, decrivez ci-dessous [ ]

CHARACTERISTIQUES DE SORTIE DE LA POUPONNIERE

Mode de paiement

☐ a) Malade lui-même ☐ b) Cie d'Assurances ☐ c) Sécurité Sociale ☐ d) Ministère de la santé

☐ e) Plan d'assurance hospitalière ☐ f) Armée / Forces Armées ☐ g) COOP ☐ h) Autre mode privé

☐ i) Autre mode publique ☐ j) Combinaison de deux

Combinaison 1 ☐ **Combinaison 2** ☐

Statut à la sortie ☐ Vivant ☐ Décédé

Si vivant, ☐ Renvoyé à la maison ☐ Transféré à un autre hôpital **Spécifier** ☐

Sortie contre avis médical ◯ Non ◯ Oui

Poids à la sortie ☐ **grams**

Age lors du décès ☐ < 24 hours ☐ 1-7 days
